# Evaluation of Illicit Drug Consumption by Wastewater Analysis Using Polar Organic Chemical Integrative Sampler as a Monitoring Tool

**DOI:** 10.3389/fchem.2021.596875

**Published:** 2021-03-30

**Authors:** Roberta Zilles Hahn, Carlos Augusto do Nascimento, Rafael Linden

**Affiliations:** ^1^ Laboratory of Analytical Toxicology, Universidade Feevale, Novo Hamburgo, Brazil; ^2^ Department of Production Engineering, Faculdades Integradas De Taquara, Taquara, Brazil; ^3^ National Institute of Forensic Science and Technology (INCT Forense), Porto Alegre, Brazil

**Keywords:** wastewater-based epidemiology, passive sampling, polar organic chemical integrative sampler, drug consumption, residual water

## Abstract

Illicit drug abuse is a worldwide social and health problem, and monitoring illicit drug use is of paramount importance in the context of public policies. It is already known that relevant epidemiologic information can be obtained from the analysis of urban residual waters. This approach, named wastewater-based epidemiology (WBE), is based on the measurement of specific markers, resulting from human biotransformation of the target drugs, as indicators of the consumption of the compounds by the population served by the wastewater treatment installation under investigation. Drug consumption estimation based on WBE requires sewage sampling strategies that express the concentrations along the whole time period of time. To this end, the most common approach is the use of automatic composite samplers. However, this active sampling procedure is costly, especially for long-term studies and in limited-resources settings. An alternative, cost-effective, sampling strategy is the use of passive samplers, like the polar organic chemical integrative sampler (POCIS). POCIS sampling has already been applied to the estimation of exposure to pharmaceuticals, pesticides, and some drugs of abuse, and some studies evaluated the comparative performances of POCIS and automatic composite samplers. In this context, this manuscript aims to review the most important biomarkers of drugs of abuse consumption in wastewater, the fundamentals of POCIS sampling in WBE, the previous application of POCIS for WBE of drugs of abuse, and to discuss the advantages and disadvantages of POCIS sampling, in comparison with other strategies used in WBE. POCIS sampling is an effective strategy to obtain a representative overview of biomarker concentrations in sewage over time, with a small number of analyzed samples, increased detection limits, with lower costs than active sampling. Just a few studies applied POCIS sampling for WBE of drugs of abuse, but the available data support the use of POCIS as a valuable tool for the long-term monitoring of the consumption of certain drugs within a defined population, particularly in limited-resources settings.

## Introduction

Abuse of licit and illicit drugs is an issue of global concern, with significant adverse impacts on human health and social welfare. Of particular concern is the abuse of illicit drugs, which are substances with prohibited or controlled nonmedical use, according to national laws (EMCDDA, 2019; UNODC, 2019). The nonmedical consumption of these controlled drugs (like cocaine, amphetamines, and Cannabis, among others) is usually associated with criminal activities, with severe social impacts (EMCDDA and Europol, 2019).

According to the World Drug Report, from the United Nations Office on Drugs and Crime (UNODC, 2019), 271 million people (5.5% of the World population between 15 and 64 years old) used illicit drugs at least once in 2017. Moreover, the same report estimates 35 million people suffering from illnesses due to drug consumption, with only one in each seven affected individuals receiving adequate treatment.

In this context, knowledge of the consumption behavior of these compounds is of utmost importance to develop damage reduction strategies and also to guide law enforcement strategic actions (Gao et al., 2018). Classical strategies to evaluate drug consumption behavior at the population level are based on epidemiological, sociological, and criminological indicators (EMCDDA, 2016). These strategies have several limitations, being intrinsically imprecise and inaccurate (Hernández et al., 2018). A novel and potentially sensitive way to detect emerging tendencies on drug abuse at a given population is the analysis of biomarkers of drug use in residual waters of a defined region, served by a wastewater treatment plant (WWTP) (van Wel et al., 2016). This evaluation approach is named wastewater-based epidemiology (WBE), which is defined as an analytical strategy to estimate drug consumption in a given population based on back-calculations, from concentrations of biomarkers measured in residual water (Devault et al., 2017b).

WBE is based on the principle that consumed drugs are excreted, either unaltered or as a mixture of metabolites, in urban wastewater networks and that the concentration of these chemical markers can be used to estimate the amount of drug consumed by the population served by the WWTP (van Wel et al., 2016; Hernández et al., 2018). WBE has the benefit of being able to detect changes in drug consumption patterns in a very sensitive and almost immediate way, being considered complementary to classical epidemiological tools (Gracia-Lor et al., 2017a).

A challenge to overcome in WBE is to obtain representative samples from residual waters. Single point sample collections (e.g., grab sampling) provide limited information due to the lack of temporal representativeness. A frequently used strategy is the use of automatic samplers, which allows the obtaining of composite samples, representative of a fixed period of time. Despite its value, automatic composite samplers are high-cost equipment and its use requires adequate facilities, including a power supply and environmental protection, which can be a limitation in resource-limited settings (Allan et al., 2006). Differently from the active sampling options, previously mentioned, passive samplers such as the polar organic chemical integrative sampler (POCIS) are a more affordable and flexible option to obtain representative samples for WBE (Alvarez et al., 2007).

This manuscript aims to review the most important biomarkers of drug consumption in wastewater, the fundamentals of POCIS sampling in WBE, the previous application of POCIS for WBE of drugs of abuse, and to discuss the advantages and disadvantages of POCIS sampling, in comparison with other strategies in WBE. To this end, the PubMed database was searched considering articles published between the years 2000 and 2020, using the following keywords, both isolated or in combination: wastewater-based epidemiology, illicit drugs, passive sampling, and POCIS. The following filters were applied: full text, journal article, review, systematic review, English, and from 2000 to 2020. The combination of the keywords wastewater-based epidemiology and illicit drugs resulted in 116 hits, whereas POCIS and illicit drugs resulted in ten hits and POCIS and wastewater-based epidemiology resulted in only one hit. After checking for duplication of data, 99 published manuscripts were reviewed, with the addition of five online documents from national and international recognized agencies.

## Wastewater-Based Epidemiology

Illicit drugs and its metabolites are emerging pollutants, and these compounds are frequently detected in environmentally relevant specimens, such as surface and residual waters (Boleda et al., 2009). As feces and urine contain amounts of ingested products, such as food, pharmaceuticals, and abused drugs, along with their metabolites, residual waters are an important source of information about the health conditions of a given population (Gracia-Lor et al., 2017a). It is also important to note that drugs can be found in residual waters as a result of accidental or intentional discharge from consumers of clandestine laboratories, making particularly relevant the use of metabolites as markers of human consumption (Pal et al., 2013). The evaluation of the presence of drugs and metabolites in environmental waters became feasible with the development of highly sensitive analytical methods (Gogoi et al., 2018). In this context, the use of biomarker concentrations in residual waters to estimate human consumption of drugs is named WBE (Causanilles et al., 2017).

The first report of the use of WBE for the estimation of illicit drug consumption dates from almost 20 years (Daughton, 2001). WBE requires knowledge of the size of the population served by the WWTP, the flow rate of the influent in the WWTP, and the metabolic rate of the parent drug with respect to the measured metabolite, along with the measured concentrations (Daughton, 2001). Recently, the European Drug Report included the use of WBE as a recommended method for monitoring illicit drug use at the population level, mainly due to the possibility of fast result reporting, almost in real-time, which allows immediate actions from the public authorities (EMCDDA, 2019).

Classical strategies to evaluate drug consumption at the population level are based on information gathered from questionnaires, drug seizing statistics, and criminal and medical records (EMCDDA, 2016). These classical approaches are dependent on the self-report of the participants of the survey. However, the reliability of the self-report is affected by moral and social restrains, which can significantly impact the quality of the data (van Wel et al., 2016). Additionally, population surveys are expensive and complex to perform (Hernández et al., 2018). The benefits of WBE resulted in the publication of studies in many countries of the world (Archer et al., 2018; Bannwarth et al., 2019; Banta-Green et al., 2016; Bartelt-Hunt et al., 2009; Baz-Lomba et al., 2016; Benaglia et al., 2020; Boleda et al., 2009; Burgard et al., 2019; Foppe et al., 2018; Kankaanpää et al., 2016; Mackulˇak et al., 2014; Maldaner et al., 2012; Metcalfe et al., 2010; van Nuijs et al., 2009b; Zhang et al., 2019; Zuccato et al., 2008). In fact, since 2011, the Europe-wide network (Sewage analysis CORe Group Europe (SCORE)) performs the systematic monitoring of consumption biomarkers of four priority drugs (cocaine, methylenedioxymethamphetamine (MDMA), amphetamine, and methamphetamine) in WWTPs, covering 68 cities from 23 European countries in 2019 (EMCDDA, 2020).

However, WBE cannot provide information on the most common administration route, profile of the consumers, or purity and quality of the used drugs. Other challenges on the application of WBE include the uncertainties on the representativeness of the sampling procedure, lack of knowledge about the stability and chemical behavior of the measured biomarkers on the residual waters, variable analytical reliability of the measurements, availability of strategies to estimate the population size served by the WWTP, and the uncertainties on the calculation procedure to retrospective estimate drug consumption by the population (Thomas et al., 2012; Castiglioni et al., 2013).

The estimation of the daily drug consumption per inhabitant (C, mg day^−1^ 1,000 inh^−1^) using WBE is based on a retrospective calculation, as presented in Eq. 1. First, the raw daily drug consumption of the drug at the population served by the WWTP is estimated by multiplying the concentration of the biomarker (c, ng L^−1^) in a representative sample by the daily influent flow at the WWTP (Q_v_, L day^−1^) and by a correction factor (f), which accounts for the average excretion rate of the biomarker and for the ratio between the molecular weight of the parent drug and its metabolite (van Nuijs et al., 2011; Zuccato et al., 2008). Afterward, the daily drug consumption per inhabitant (inh) is obtained dividing by the number of individuals served by the WWTP. The value is multiplied by 1,000 to normalize for 1,000 inhabitants. Table 1 presents an overview of *f* values described in previous studies. Important to note is that these calculations require that the measured biomarker is specific and unique for a certain drug (Zuccato et al., 2008).

$$C=\left[\frac{c*Qv*f}{inh}\right]*1000$$ [1]

Besides the correction applied in Eq. 1, the measured concentrations can also be multiplied by a correction factor that takes the biomarker stability on residual waters into account. van Nuijs et al., 2011 considered that ecgonine methyl ester (EME), amphetamine, and 6-monoacetyl morphine (6-MAM) had a degradation percentage of 20, 30, and 30%, respectively, during their residence time in the wastewater. Then, the authors used a stability correction factor of 1.25, 1.43, and 1.43 for EME, amphetamine, and 6-MAM, respectively. Compounds presenting minimal degradation, like benzoylecgonine (BZE), methamphetamine, MDMA, and 2-ethylidene-1,5-dimethyl-3,3-diphenylpyrrolidine (EDDP), did not require the use of a stability correction factor (van Nuijs et al., 2011).

**TABLE 1:** Target compounds, biomarkers, excretion rates, and correction factors used in retrospective consumption calculations on the context of WBE.

Compound	Biomarker	Excretion rate of the biomarker (%)	Molecular weight ratio between drug and biomarker	Correction factor (f)	References
Cocaine	BZE	45	1.05	2.33	Daglioglu et al. (2019), Devault et al. (2014), Fallati et al. (2020), Foppe et al. (2018), Maldaner et al. (2012), Postigo et al. (2010), Postigo et al. (2011), van Nuijs et al. (2009a), Zuccato et al. (2005), Zuccato et al. (2008)
		38	1.05	2.77	Thomas et al. (2012), Mackulak et al. (2014), Mackuľak et al. (2019)
		35	1.05	3.0	van Nuijs et al. (2011)
		35	1.10	3.14	Lai et al. (2011), Lai et al. (2013)
		30.07	1.05	3.49	Baker et al. (2014)
		30	1.05	3.50	Zhang et al. (2019)
		29	1.05	3.59	Castiglioni et al. (2013), Causanilles et al. (2017), Mercan et al. (2019), Ort et al. (2014b), van Wel et al. (2016)
		29	1.05	3.62	Archer et al. (2018)
	Cocaine	7.5	1.00	13.33	Lai et al. (2011)
		1.53	1.00	65.36	Baker et al. (2014)
	EME	15	1.52	10.20	van Nuijs et al. (2011)
	NBZE	0.95	1.10	115.79	Baker et al. (2014)
	Norcocaine	0.037	1.05	2,837.84	Baker et al. (2014)
Crack	AEME	0.19	1.67	878.95	Baker et al. (2014)
Amphetamine	Amphetamine	36	1	2.77	Krizman-Matasic et al. (2019), Mercan et al. (2019)
		30	1	3.33	Baker et al. (2014), Daglioglu et al. (2019), Devault et al. (2014), Emke et al. (2014), Fallati et al. (2020), Foppe et al. (2018), Postigo et al. (2010), Postigo et al. (2011), van Nuijs et al. (2011), van Wel et al. (2016), Zuccato et al. (2008)
Metamphetamine	Metamphetamine	43	1	2.33	Archer et al. (2018), Baker et al. (2014), Daglioglu et al. (2019), Fallati et al. (2020), Foppe et al. (2018), Postigo et al. (2010), Postigo et al. (2011), van Nuijs et al. (2011), Zhang et al. (2019), Zuccato et al. (2008)
		39	1	2.56	Lai et al. (2011)
		33	1	4.06	Lai et al. (2013)
		22.7	1	4.41	Mercan et al. (2019)
	Amphetamine	5.5	1.1	20.1	Lai et al. (2011), Archer et al. (2018)
	Norephedrine	5.0	0.99	19.7	Archer et al. (2018)
MDMA	MDMA	65	1	1.54	Zuccato et al. (2008), Postigo et al. (2010), Devault et al. (2014), van Wel et al. (2016), Daglioglu et al. (2019), Fallati et al. (2020)
		26	1	3.85	Postigo et al. (2011), Foppe et al. (2018)
		22.5	1	4.44	Archer et al. (2018), Krizman-Matasic et al. (2019), Mercan et al. (2019)
		20.3	1	4.93	Baker et al. (2014)
		20	1	5.0	van Nuijs et al. (2011), Zhang et al. (2019)
		15	1	6.67	Lai et al. (2011), Emke et al. (2014)
	HMMA	18.2	0.99	5.0	Archer et al. (2018)
MDEA	MDEA	19	1	5.26	Baker et al. (2014), Foppe et al. (2018)
Heroine	Heroin	0.025	1	4,000	Baker et al. (2014)
	Morphine	55	1.29	2.35	Baker et al. (2014)
		42.5	1.29	3.04	Daglioglu et al. (2019)
		42	1.29	3.07	Zuccato et al. (2008), Boleda et al. (2009), Postigo et al. (2010), Fallati et al. (2020)
		4.2	1.29	30.71	Foppe et al. (2018)
	6-MAM	1.3	1.13	86.92	Postigo et al. (2011), van Nuijs et al. (2011), Foppe et al. (2018), Krizman-Matasic et al. (2019), Fallati et al. (2020)
		0.5	1.13	226	Baker et al. (2014)
Morphine	Normorphine	5	1.05	21.0	Baker et al. (2014)
Codeine	Codeine	63.8	1	1.57	Baker et al. (2014)
		30	1	3.33	Zhang et al. (2019)
	Norcodeine	5.1	1.05	20.59	Baker et al. (2014)
THC	THCCOOH	2.5	0.91	36.4	Postigo et al. (2011)
		0.6	0.91	100	van Wel et al. (2016), Daglioglu et al. (2019)
		0.6	0.91	152	Zuccato et al. (2008), Boleda et al. (2009), Postigo et al. (2010), Lai et al. (2011), Devault et al. (2014), Mercan et al. (2019), Fallati et al. (2020)
		0.5	0.91	182	Causanilles et al. (2017), Foppe et al. (2018), Krizman-Matasic et al. (2019)
Ketamine	Ketamine	20	1	5.0	Du et al. (2020)
		2.3	1	43.48	Baker et al. (2014)
	Norketamine	4	1.06	26.50	Du et al. (2020)
		1.6	1.06	65	Lai et al. (2013)
		1.6	1.06	66.25	Baker et al. (2014), Zhang et al. (2019)
Phencyclidine	Phencyclidine	10	1	10	Baker et al. (2014)
Methadone	Methadone	27.8	1	3.60	Baker et al. (2014)
		27.5	1	3.64	Postigo et al. (2011)
	EDDP	55	1.06	1.93	Du et al. (2019), Zhang et al. (2019)
		25	1.12	3.6	Krizman-Matasic et al. (2019)
		25	0.82	3.28	Boleda et al. (2009)
		24.6	1.06	4.31	Baker et al. (2014)
		23	1.12	4.87	van Nuijs et al. (2011)
		13	0.82	6.31	Devault et al. (2014)
Mephedrone	Mephedrone	15.4	1	6.5	Archer et al. (2018)
Mescaline	Mescaline	57.5	1	1.74	Baker et al. (2014)
Ephedrine	Ephedrine	75	1	1.33	Postigo et al. (2010), Postigo et al. (2011)

6-MAM, 6-monoacetylmorphine; AEME, anhydroecgonine methyl ester; BZE, benzoylecgonine; EDDP, 2-ethylidene-1,5-dimethyl-3,3-diphenylpyrrolidine; EME, ecgonine methyl ester; HMMA, 4-hydroxy-3-methoxymethamphetamine; MDEA, methyldiethanolamine; MDMA, 3,4-methylenedioxy-N-methylamphetamine; NBZE, norbenzoylecgonine; THC, Δ⁹-tetrahydrocannabinol; THCCOOH, 11-nor-9-carboxy-THC; WBE, wastewater-basedepidemiology.

## Biomarkers of Drug Consumption in Residual Waters

The measured biomarkers in WBE are preferentially specific metabolites of the drug of interest with elimination mainly by the renal route, with wastewater concentrations in the range of ng L^−1^ or higher (Gracia-Lor et al., 2017a; Vazquez-Roig et al., 2013). In addition to these characteristics, the biomarkers must have acceptable stability in wastewater since their entrance into the sewage system until sampling for analysis, storage, and processing (McCall et al., 2016). The removal of the biomarkers from wastewater can be attributed to chemical modifications on the water environment, as well as to microbiological biotransformation (Mardal and Meyer, 2014) and adsorption to particulate matter present in the sewage system and in the WWTP (Daughton, 2012; McCall et al., 2016). In fact, the knowledge of the stability of a certain biomarker at their environmental exposure conditions is mandatory before the use of concentration data in WBE, with a significant impact on the overall uncertainty of drug consumption estimation (Castiglioni et al., 2013). Laboratory simulation studies are often used to evaluate the stability of the biomarkers at different pH and temperature conditions, which also can modify microbiological activity, trying to simulate the actual conditions (Devault et al., 2017a). Adsorption to particulate matter present at the sewage and WWTP can be simulated using fortified residual waters and also evaluated at a realistic range of pH values and temperatures (Devault et al., 2017a).

## Cocaine

COC is the main psychoactive alkaloid present in *Erythroxylum coca* leaves. After intake, COC is hydrolyzed in the liver mainly to BZE and EME, which are excreted in urine at an average of 45 and 40% of the administered dose, respectively (Baselt, 2000). Cocaethylene (CE) is also formed by biotransformation when COC is used in combination with ethanol. Norcocaine (NCOC) is a minor oxidative metabolite. COC is used mainly as its chloridrate, by intravenous and intranasal routes, or as the free base (crack cocaine), by the respiratory route. When the free base is smoked, pyrolytic metabolites are formed, such as anidroecgonidine and anidroecgonidine methyl ester (Feitosa et al., 2013). The biomarker of COC most frequently used in WBE studies is BZE. Differently from COC, BZE is highly stable in residual waters. However, it is important to note that BZE can also be formed from COC degradation at residual waters, which can result in an overestimation of COC consumption if this conversion is not taken into account (Plósz et al., 2013). The literature reported COC excretion rates in the range of 1–9% for the parent drug and about 45% for BZE (Baselt, 2000). Considering these average excretion rates, COC to BZE concentration ratios in residual waters in the range of 0.02–0.2 are an indication of drug consumption in the population served by the WWTP, whereas higher ratios can be suggestive of other COC sources, like leakages from clandestine laboratories (Castiglioni et al., 2011). However, as the COC to BZE ratio can also be affected by the temperature, complementary studies are needed to establish a cut-off ratio for the classification of the source of COC in the sewage system (van Nuijs et al., 2009b). In fact, the possible presence of COC in residual waters from nonhuman sources limits its use of a marker of drug consumption (van Nuijs et al., 2011).

Both BZE and EME concentrations in residual waters can be used for the estimation of COC consumption, usually resulting in similar results. However, the use of BZE is preferred due to its higher stability in water (van Nuijs et al., 2011). The most frequently reported correction factor for the estimation of COC consumption using BZE concentrations in residual waters is 2.33, which considers that BZE mounts to 45% of excreted COC (Daglioglu et al., 2019). This correction factor does not consider the simultaneous consumption of COC with other substances, particularly ethanol. The fraction of COC excreted as BZE and EME is significantly reduced when the drug is used along with ethanol due to the formation of CE (Harris et al., 2003). As the simultaneous use of COC and ethanol is common, van Nuijs et al., 2011 employed a correction factor of 3.0 for BZE concentrations, estimating that 35% of COC is excreted as BZE in this condition. If EME concentrations in residual waters are used for the estimation of COC consumption, a correction factor of 10.2 was proposed, which considers that 15% of the COC dose is excreted as EME (van Nuijs et al., 2011).

Castiglioni et al. (2006) evaluated the stability of illicit drugs and metabolites in residual water by analyzing laboratory prepared solutions in amber vials stored at 4ºC for three days. In these conditions, the concentrations of COC, CE, and NCOC were reduced on 36, 15, and 13%, respectively. These concentration reductions were in parallel with the increase in the concentrations of the metabolites BZE and norbenzoylecgonine (NBZE).

## Opiates

The opiate group of drugs includes not only prescription pharmaceuticals, like fentanyl, oxycodone, morphine, codeine, and tramadol, but also illicit compounds like heroin. The majority of the opiate drugs and metabolites are rapidly decomposed at residual waters. Additionally, several opiates are decomposed or metabolized to morphine, which presents some level of stability on the sewage. Therefore, by measuring morphine levels only, it is not possible to estimate the drug consumption (Werschler and Andrew, 2019).

Morphine is excreted in the urine mainly as morphine-3-βD-glucuronide. As this compound is usually found at a very low concentration in residual water, deconjugation is likely to happen due to the enzymatic activity of bacteria present on sewage (Castiglioni et al., 2006). The contribution of codeine consumption to the morphine levels found on residual water is considered to be insignificant, once morphine is a minor metabolite of codeine (Baselt, 2000). The estimation of heroin consumption by WBE using morphine as a biomarker must consider the potential contribution of therapeutic drugs to the measured concentrations (Zuccato et al., 2008). Alternatively, 6-MAM can be used as the biomarker of heroin consumption in residual water due to the higher specificity. However, the high value of the correction factor can lead to significant uncertainties (van Nuijs et al., 2011).

## Cannabis

The main psychoactive compound from the marijuana plant, *Cannabis sativa*, is tetrahydrocannabinol (THC). THC is metabolized by hydroxylation, forming the main active metabolite 11-hydroxy-tetrahydrocannabinol (11-OH-THC) and the minor metabolite 8-beta-hydroxy-tetrahydrocannabinol (8-β-OH-THC). The further oxidation of 11-OH-THC produces the main inactive metabolite 11-nor-9-carboxy- tetrahydrocannabinol (THC-COOH) (Baselt, 2000). As the conversion of 11-OH-THC to THC-COOH is very fast, the latter is the most commonly used biomarker for the retrospective calculation of THC exposure in WBE. THC-COOH is excreted in urine and feces as a glucuronide conjugate, being hydrolyzed by *β*-glucuronidases present on fecal bacteria present in untreated residual water (Castiglioni et al., 2006). However, only a small amount of THC is excreted in the form of THC-COOH, requiring sensitive analytical methods for its detection. THC-COOH is a specific metabolite of THC, and the concentration of this biomarker is not affected by the use of other drugs in the population of the study (Werschler and Andrew, 2019).

Variable values of *f* were reported for the estimation of THC consumption from THC-COOH concentrations in WBE studies, as presented in Table 1. Gracia-Lor et al. (2016) and Huestis et al. (1996) established excretion rates of 0.5-0.6% considering the consumption of smoked marijuana. Alternatively, Postigo et al., 2011 used a higher excretion rate, of 2.5%, considering that all excreted 11-OH-THC was oxidized *in situ* to THC-COOH. Currently, the partition behavior of THC-COOH between water and particulate matter, present on the sewage system and WWTP, is not completely known, which can result in significant errors on the estimation of the mass of THC used by a given population (Causanilles et al., 2017).

## Amphetamine Stimulants

The amphetamine stimulant group includes amphetamine itself and its derivatives, like methamphetamine, and ecstasy-like compounds, like 3,4-methylenedioxyamphetamine (MDA), 3,4-methylenedioxy-N-ethylamphetamine (MDEA), and MDMA, among others. Differently of COC, amphetamine-type drugs are excreted mainly as the parent drugs. This characteristic of the consumption biomarkers in residual water can be a limitation to the identification of the presence of the raw drugs on the sewage system. However, most of the amphetamine compounds are racemic compounds, and the result of chemical synthesis has equal proportion of both enantiomers. However, the molecules formed after biotransformation will result in a particular enantiomeric proportion (Kasprzyk-Hordern and Baker, 2012; Emke et al., 2014). In this context, the characterization of the enantiomeric profile of the biomarkers can be used to differentiate between population consumption of the drug and disposal of the raw material on the sewage (Archer et al., 2018). A correction factor of 1.5 was originally proposed by Zuccato et al., 2008 for the estimation of MDMA consumption after measuring the concentration of the parent drug in residual water. This correction factor considers an excretion rate of 65% of the used dose as the parent compound. However, a more recent study showed that only 15% of the used dose is actually excreted as MDMA, and a correction factor of 6.67 should be used (Abraham et al., 2009).

A laboratory study of the stability of amphetamine, methamphetamine, MDA, MDEA, and MDMA found a maximum degradation rate of 5% (Castiglioni et al., 2006).

## Other Compounds Evaluated in Previous Studies

Methadone, a synthetic opioid drug used as an analgesic and heroin-substitution treatment, was already studied in WBE. The used biomarker is EDDP (Du et al., 2019). Ketamine, abusively used due to its dissociative and hallucinogenic effects, was also evaluated in WBE studies, using both ketamine and the metabolite norketamine as biomarkers (Baker et al., 2014). These authors reported the use of excretion rates of 1.6% and 2.3% for ketamine and norketamine, respectively. Recently, Du et al., 2020 concluded that excreted rates estimated based on pharmacokinetic studies were not appropriate for ketamine and suggested a much higher excretion factor, of 20%, relying on data from local drug seizures. Other compounds like mephedrone, mescaline, and ephedrine were also evaluated.

## Biomarkers of Population Size in Residual Waters

The estimation of drug consumption by a population served by a WWTP requires knowledge of the size of this population (Eq. 1). Census data can be outdated, leading to erroneous estimations. Different strategies were proposed to estimate the size of a population served by a WWTP, and the combination of estimation approaches is recommended to avoid deviations associated with a given method. Classical approaches include the designed capacity of the WWTP, census data, and hydro-chemical measurement parameters (Castiglioni et al., 2014). The design capacity of the WWTP is usually not reliable to estimate the population size once the plant can operate either above or below its projected capacity. Census data are not adjusted over time and do not take into account seasonal population changes, as a result of tourism and other population movements. Population size estimations can also be made using hydro-chemical parameters such as chemical oxygen demand (COD), biological oxygen demand (BOD), total nitrogen (N), and total phosphorus (P) (van Nuijs et al., 2011). Another valuable strategy is to measure concentrations of anthropogenic markers in residuals waters, like human endogenous compounds or metabolites of widely consumed products, caffeinated beverages, and tobacco cigarettes, among others.

The number of inhabitants served by a WWTP can be estimated using phosphorus, nitrogen, BOD, and COD levels on the residual waters, considering that a single person releases the equivalent to 1.7 g day^−1^ of phosphorus, 12.5 g day^−1^ of nitrogen, 59 g day^−1^ of BOD, and 128 g day^−1^ of COD (van Nuijs et al., 2011). This approach was applied to a WWTP located at Brussels, Belgium, and a wide range of served inhabitants was estimated along two consecutive months, between March 2009 and January 2010, with values in the range of 77.831 to1.670.562, contrasting to the WWTP capacity of 1.1 million inhabitants. It is important to note that some variation in the number of served inhabitants is expected, once it is affected by several factors, like holiday periods and the occurrence of large public events. These results demonstrate that the use of WWTP capacity as the number of inhabitants served in the sewage epidemiology does not reflect the actual number of inhabitants served and should be replaced by real-time calculations of these parameters (van Nuijs et al., 2011). However, these hydro-chemical parameters do not only reflect human metabolism but also the presence of other biodegradable substances in the sewage system, being affected by industrial leakages, agricultural activities, and disposal of food residues, among others (Daughton, 2012).

Anthropogenic markers of population size in residuals waters must fulfill some requisites: present a predictable and constant elimination in urine, high stability in residual water, and be of exclusive human origin. Several potential candidate anthropogenic biomarkers were proposed, particularly creatinine, cotinine, and coprostanol (Daughton, 2012).

Senta et al. (2015) employed nicotine metabolites as population size biomarkers and found a good agreement with census data in Como, Italy. However, a limitation of the use of these markers is the need for a constant number of smokers throughout the investigated populations. Rico et al. (2017) evaluated twelve different urinary biomarkers as indicators of population size and found a similar population size when the estimation was made using either cotinine, 5-hydroxyindoleacetic acid, and caffeine compared with the hydro-chemical parameters (Rico et al., 2017).

Other alternative approaches for the estimation of the size of a population served by a WWTP have been described. Thomas et al. (2017), in a study performed in Norway, used data from a local mobile phone provider to estimate the population present in a given service area and used this population size to estimative the drug consumption in a dynamic way, particularly during the holiday period (Thomas et al., 2017).

A variety of urinary markers, derived from pharmaceuticals and personal care products, were evaluated in residual water in Australia, along with the population census of 2011 and with the per capita consumption of selected products, provided by the Australian Government through the Pharmaceutical Benefit Scheme (O’Brien et al., 2014). The concentration of the makers atenolol, carbamazepine, codeine, furosemide, gabapentin, hydrochlorothiazide, ibuprofen, naproxen, norfloxacin, paracetamol, acesulfame, and caffeine presented high correlation (*r* ^2^ > 0.8) with the population size.

Caffein itself is considered to be a potentially biased biomarker of population size, once it comes not only from drinking coffee but also from other sources like coffee grounds spilled in the sink drain. However, 1,7-dimethyluric acid is a specific human caffeine metabolite, formed from paraxanthine (Gracia-Lor et al., 2017b). Then, considering the widespread human consumption of caffeine, 1,7-dimethyluric acid could be used as a biomarker to chemically estimate the population size of a population served by a WWTP.

The size of the population (inh) served by the WWTP can be estimated using Eq. 2, using the concentration of anthropogenic biomarkers in residual water. In this equation, C_ab_ is the concentration of the anthropogenic biomarker, Qv is the daily influent flow at the WWTP (Qv, L day^−1^), ER is the excretion rate of the biomarker, and DDD is the defined daily dose of the parent compound of the biomarker (mg per 1,000 inhabitants) (Rico et al., 2017). DDD data can be obtained from average selling data of the parent drug in the region served by the studied WWTP.

inh=(Cab∗Qv∗ER)/DDD [2]

An important methodological advantage of the use of anthropogenic biomarkers for the estimation of population size is that biases at influent flow measurements are neutralized at the retrospective calculation of drug consumption, using Eq. 1 (Lai et al., 2011).

## Sampling Strategies for the Estimation of Drug Consumption Biomarkers in Residual Water

Residual water collection at a WWTP in the context of drug consumption estimation must be representative of the 24 h of the day (Ort, 2014). One of the limitations of the use of WBE for drug consumption estimation is associated with the limited temporal representativeness, which must be taken into consideration during data interpretation (Baz-Lomba et al., 2016).

Many previous studies of WBE were limited to one-week sampling schemes (Ort et al., 2014b). However, stratified random sampling schemes (56 specimens per year) are recommended to estimate a representative average annual consumption of drugs (Ort et al., 2014a). Ort et al. (2014a) reported an annual average estimation error of COC consumption of 60% when only seven consecutive day samples of residual water were analyzed. This difference was attributed to the temporal variation of the drug consumption behavior by the population served by the WWTP. However, when 56 stratified random collected samples were tested, the deviation is expected to be around 10%. Increasing sampling frequency can lead to higher costs, also requiring a continuous supply of energy and availably of physical space for the sampling equipment. A higher sampling frequency will not be adequate when the patterns of drug use are rapidly changing or the concentrations are affected in the short term by external factors, as rain precipitation (Ort et al., 2014a). Automatic sampling devices are programmed to collect several sample aliquots during the 24 h of the day, keeping the aliquoted specimens in a refrigerated compartment. The representativeness of the composite samples obtained with this kind of device is dependent on the minimal sample volume that can be collected, the storage capacity of the device, and its incapacity to account for high flow events, such as abundant rain. Usually, the composite sample has a 1–20 L volume, and a subsample can be directly analyzed or submitted to an extraction procedure (Ort et al., 2010).

Passive sampling devices (PSDs) are an alternative sampling strategy used to overcome some of the limitations described above. Particularly, PSDs are useful tools for screening and long-term monitoring of the use of drugs in WBE (Baz-Lomba et al., 2017), in a more straightforward and economical way when compared with spot or composite sampling (Allan et al., 2006; Magi et al., 2018). Additionally, PSDs are less affected by short-term variations in the concentration of drug consumption biomarkers (Harman et al., 2011; Kaserzon et al., 2014). As described by Baz-Lomba et al., 2017, annual drug monitoring in wastewater can be estimated using a relatively small number of passive samplers (*n* = 24). Passive sampling combines both sampling and preconcentration of the compounds of interest in a single step (Magi et al., 2018). This characteristic allows the achievement of lower limits of detection than classical spot sampling or active sampling, once the *in situ* exposure occurs for several days (Morin et al., 2013). As an example, Fedorova et al., 2014 found several drug biomarkers in a PSD extract (BZE, ketamine, methadone, and midazolam) which were not detected in spot samples. PSD allows the estimation of time-weighted average (TWA) concentrations, in an economical and robust way, being of easy implementation at the point of collection, without the need for specific and sophisticated equipment and energy source (Alvarez et al., 2004).

## Polar Organic Chemical Integrative Sampler

**FIGURE 1:**
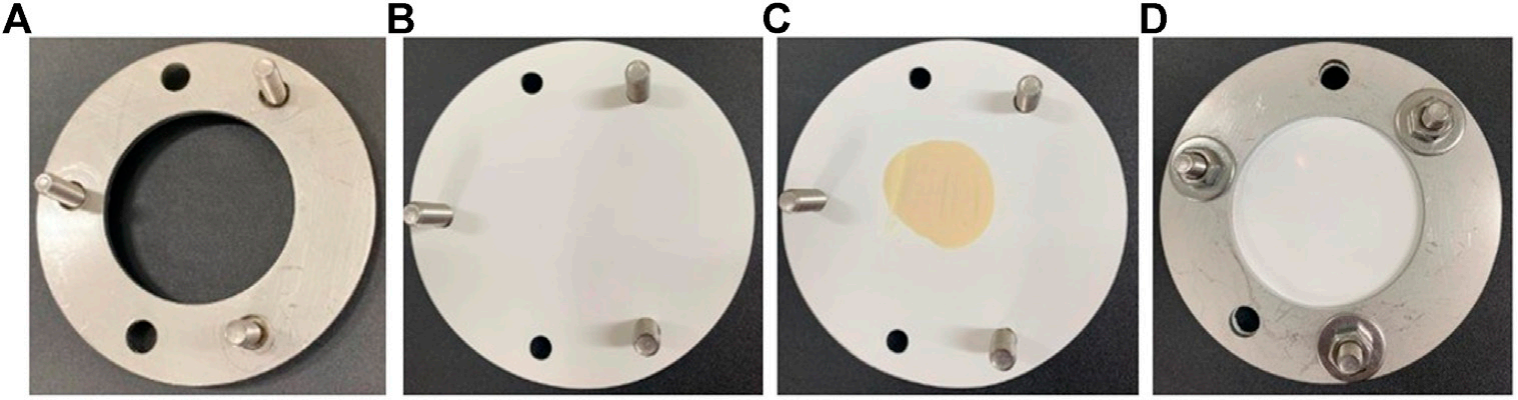
POCIS assembly. **(A)** The bottom compression washer is assembled with screws. **(B)** PES membrane placed over the lower compression washer. **(C)** The amount of 200 mg of the sorbent is placed in the center of the PES membrane. **(D)** A second PES membrane is placed over the sorbent and the upper compression support washer is added. The screws and nuts are tightened to secure the support and prevent the loss of the solid sorbent.

Among the available PSD, the POCIS has been used for monitoring concentration of hydrophilic compounds, such as pesticides, pharmaceutical, and personal care products (Kaserzon et al., 2014). POCIS was introduced by Alvarez et al., 2004 and consists of sorbent material sandwiched between two polyethersulphone (PES) membranes. POCIS is usually built using physically resistant materials as a structural basis, like stainless steel or aluminum. Two structural washers are used to compress two PES membranes, with the sorbent material being sandwiched between the membranes. The whole structure of the device is fixed with screws. The original study of Alvarez et al., 2004 employed washers and PES membranes of 90 mm of diameter, resulting in a membrane chemical exchange area of ≅ 41 cm^2^. The structure of a laboratory-made POCIS is presented in Figure 1.

Usually, the POCIS is immersed for more than one week in water and accumulates the sampled compound by passive diffusion (Morin et al., 2013). The microporous PES membrane acts as a semipermeable barrier between the sorbent and the external environment, allowing the diffusion of organic polar solutes to the sorbent while avoiding that particulate matter, colloids, and microorganisms (with diameters higher than the membrane pore, usually 0.1 µm) pass through the membrane (Alvarez et al., 2004).

The original study of Alvarez et al., 2004 evaluated different membrane composition and selected PES due to the combination of high analyte uptake rates, minimal biological incrustation, and high durability on the sewage environment. The composition of the sorbent in POCIS depends on the target compounds to be sampled, and the most commonly used are named pesticide POCIS (Pest-POCIS) and pharmaceutical POCIS (Pharm-POCIS). Pest-POCIS sorbent is a mixture of three solid-phase sorbents: Isolute ENV+, polystyrene divinylbenzene, and Ambersorb 1,500 carbon. Pest-POCIS is used for monitoring concentrations of most pesticides, hormones, and several other chemicals. The Pharm-POCIS sorbent contains only the solid-phase extraction sorbent Oasis HLB® and is used for the sampling of pharmaceutical compounds and its metabolites in water. Oasis HLB® is a copolymer of [(poly [divinylbenzene]-co-N-vinylpyrrolidone)] and provides analyte retention based on hydrophilic-lipophilic balance retention, with the capacity of retaining compounds with a wide range of polarities (Alvarez et al., 2004). Both Pest-POCIS and Pharm-POCIS are commercially available and can also be prepared in house.

POCIS sampling is usually performed over several weeks, allowing the concentration of a large volume of water and accumulates the effects of periodic events that can be missed with grab sampling (Morin et al., 2013). As a result of the long exposure time of POCIS to the residual water, a TWA concentration can be obtained (Alvarez et al., 2004). The amount of the compounds at the sorbent of the POCIS after the sampling time is related to the concentration present in the water along the sampling period and is dependent of the sampling rate (R_S_), which represents the volume of water cleared of the compound by the POCIS over a given time (Magi et al., 2018).

Criquet et al. (2017) compared POCIS and composite automatic sampling for the determination of 46 pesticides and 19 pharmaceuticals in an urban river, with 2-week sampling campaigns. The authors reported a good agreement between both sampling methods, with 75% of measurements presenting ratios between 0.33 and 3. Concentrations measured with POCIS were, in general, between the maximum and minimum levels measured in the composite samples. Bishop et al. (2020) compared POCIS and composite automatic sampling for the measurement of the concentrations of drugs of abuse and pharmaceuticals in the influent of a WWTP. These authors reported a subestimation of concentrations using POCIS when compared with the median concentration of the composite sampling, with only 48% of the concentrations within a three-fold difference. However, the occlusion of the POCIS membrane, reported by the authors, could have affected the performance of the sampling device. When compared with composite automatic sampling, POCIS allows similar findings with a smaller number of samples, with cleaner sample extracts and easier handling, once large volumes of water are not needed. Besides, POCIS sampling can avoid missing a peak concentration event, which can happen if composite automatic sampling is used.

To calculate TWA concentrations, R_S_ of the analytes of interest must be established in calibration studies, which can be performed both *in situ* or in laboratory conditions (Harman et al., 2012; Morin et al., 2012). The lack of standardized R_S_ and the use of proper exposure corrections due to the influence of environmental factors are the main issues related to the estimation of TWA concentrations using POCIS (Baz-Lomba et al., 2017).

Other than POCIS, alternative PSDs were described, like those based on diffusive gradients in thin films (DGT) (Guo et al., 2017) and microporous polyethylene tubes (MPTs) (McKay et al., 2020). While these reports described the measurement of drugs of abuse concentrations, no WBE estimation was made.

## Accumulation Kinetics in Polar Organic Chemical Integrative Sampler and Determination of Sampling Rates

The accumulation of target compounds at the sorbent phase of the POCIS obeys the first-order kinetics, with an initial linear stage, followed by curvilinear and equilibrium regime (Morin et al., 2012). The accumulation of a chemical in the POCIS is described by Eq. 3, where C_S_ (ng g^−1^) is the concentration of the compound on the sorbent phase, C_W_ (ng L^−1^) is the average concentration of the compound at the residual water, K_u_ is the uptake rate of the analyte in the sorbent phase (L g^−1^ day^−1^), K_e_ is the elimination rate constant of the chemical from the sorbent phase, and t (days) is the exposure time (Morin et al., 2013).

$$Cs=Cw*\frac{K_{u}}{K_{e}}*\left(1-e^{-Ket}\right)$$ [3]

POCIS is considered as an infinite collector of contaminants and, assuming constant concentrations, the compounds are accumulated linearly within time (Alvarez et al., 2007). In this context, K_e_ is insignificant when comparing with K_u_, allowing the simplification of Eq. 3, which relates the concentration of the compound on the sorbent phase of the POCIS to the TWA concentration on the water (C_W_, ng L^−1^) through the value of the sampling rate (R_S,_ L day^−1^), as presented in Eq. 4. In this equation, M_S_ (g) is the mass of sorbent present on the POCIS and t (days) is the exposure time (Baz-Lomba et al., 2017).

$$Rs=\frac{\left(Cs*Ms\right)}{\left(Cw*t\right)}$$ [4]

When C_S_*M_S_ (the amount of contaminant accumulated in POCIS, ng) is plotted as a function of t (day), the slope of the obtained curve is C_W_*R_S_. Thus, R_S_ can be determined by dividing the slope by C_W_ (Jacquet et al., 2012).

Some authors rewrote Eq. 4 and used a concentration factor (CF, L g^−1^) to neutralize the effect of C_W_ variations, dividing the concentrations in the sorbent and in water (C_S_/C_W_), as presented in Eq. 5 (Morin et al., 2013; Baz-Lomba et al., 2017).

$$CF=\left(\frac{Cs}{Cw}\right)=\left(\frac{Rs*t}{Ms}\right)$$ [5]

The time to achieve half of the equilibrium concentration (half-time, t_1/2_) reflects the limit between the linear and curvilinear regimen (Alvarez et al., 2007). This time can be estimated through the first-order curves adjusted to the calibration data in order to confirm adsorption linearity during the exposure time (Baz-Lomba et al., 2017). Therefore, R_S_ values must be calculated during a time equal or smaller to t_1/2_ for better accuracy (Morin et al., 2013). Half-time values are calculated using Eq. 6. The value of k_e_ is usually estimated by fitting exponential curves, using specialized statistical software.

$$t_{\frac{1}{2}}=\frac{0.693}{K_{e}}$$ [6]

Morin et al. (2013) evaluated the adsorption kinetics of 56 organic micropollutants to Pharm-POCIS. Among the tested compounds, 43 have curvilinear adsorption kinetics, allowing the use of Eq. 5 to calculate R_S_ values, if exposure time was lower than t_1/2_. For these compounds, CF was calculated using C_S_ and C_W_ values obtained at different adsorption times. Afterward, the plot of CF *vs*. time allowed the obtention of a straight line, whose slope was R_S_/M_S_. From this slope value, accurate R_S_ values could be calculated, using the average weight of the POCIS sorbent exposed at the water until t_1/2_.

Half-time is an important parameter to estimate the ideal sampling time of the POCIS to obtain TWA concentrations of a given chemical. TWA (C_W_) concentrations can be calculated rearranging Eq. 4, as presented in Eq. 7, for analytes presenting t_1/2_ higher than the sampling time, once these compounds are linearly accumulated during *in situ* sampling (Morin et al., 2013).

$$Cw=\frac{\left(Cs*Ms\right)}{\left(Rs*t\right)}$$ [7]

Equation 7 is valid to estimate C_W_ when sampling is performed during the linear adsorption period. To this end, the duration of the linear regimen must be established for each monitored compound (Fedorova et al., 2014). The POCIS device should not be immersed in the sampled water for a time longer than t_1/2_. Otherwise, nonreliable estimations of TWA will be calculated (Morin et al., 2013).

POCIS is usually used in a linear regimen for the estimation of TWA concentrations with acceptable accuracy. Alternatively, POCIS can be immersed in residual water only for the screening of micropollutants, independently from the regimen, once only qualitative information is desired (Morin et al., 2012).

## Polar Organic Chemical Integrative Sampler Calibration

TWA concentrations can be calculated using R_S_ values obtained *in situ*. However, this approach requires that field calibrations are performed in each sampling campaign (Jacquet et al., 2012). Moreover, in this particular case, the contaminants must be present in the aquatic environment in a relatively constant concentration. The *in situ* calibration allows the obtention of R_S_ values specific of a certain collection location and takes into account the physicochemical conditions of the local environment (Harman et al., 2011).

Another alternative for the determination of R_S_ values is the laboratory calibration of the POCIS devices, which can be performed only once for a given compound. Laboratory calibration is more cost-effective. A potential disadvantage of laboratory calibration is that environmental conditions are not taken into consideration, which can lead to biased TWA estimations (Fedorova et al., 2014; Miller et al., 2016). Besides, it is also important to control important physicochemical parameters in water that may influence R_S_ values, such as temperature, flow, pH, conductivity, dissolved organic carbon (DOC), and the expected concentration of the compounds of interest on the water (Morin et al., 2012). Laboratory calibration is more commonly applied due to its simplicity and can be performed in both static or recirculation approaches (Arditsoglou and Voutsa, 2008; Harman et al., 2009).

The *in situ* calibration of R_S_ values of POCIS was applied to illicit drugs by Baz-Lomba et al. (2017). Accumulation curves, relating CF (C_S_/C_W_) of the compound (*y* axis) to the POCIS exposure time to the investigated residual water environment (*x* axis, in days), were fitted for exposure times of 14, 21, and 28 days. From these curves, R_S_ values were calculated as the slope of the linear part of the fitted curves for the compound of interest, forcing this curve through the origin. The average coefficient of variation (CV%) for the different *in situ* calibration sets was smaller when using the results from the first 14 days of exposure, with an average CV% lower than 17.1% for the investigated compounds. COC, BZE, morphine, and methamphetamine presented linear incorporation profiles. However, the *in situ* calibration required a parallel composite collection of water samples for the estimation of R_S_, which is required for the establishment of CF values (C_S_/C_W_, L g^−1^), as presented in Eq. 5.

Laboratory calibration can be performed using static calibration procedures or continuous flow systems. Static calibration (closed system, with analyte spiking at the beginning of the experiment) is considered to be appropriate when the compounds of interest are not rapidly degraded or adsorbed and the calibration time is smaller than one week, to reduce the influence of other processes affecting dissipation (Magi et al., 2018). The R_S_ value in laboratory calibration is calculated similarly to *in situ* calibration, but, as the water concentration of the compounds is controlled, there is no need for active composite sampling during these experiments. Another way to estimate R_S_ of a compound is to measure the decrease in the analyte concentration in water along time in a static calibration, as applied by Yargeau et al. (2014). These authors calculated R_S_ using a linear regression describing the loss of the compound from water as the result of the adsorption into the POCIS during the 8 days of the calibration experiment. In this regression, the natural logarithm of the concentrations (*y* axis) was plotted against the adsorption time (*x* axis). At the end of the calibration experiment, the POCIS was removed from the testing vessel and analyzed to compare the accumulation of the compound at the sorbent with the R_S_ calculated considering the loss of the analyte in the water. The results of this evaluation concluded that the adsorption of the compound by the PES membrane has a negligible effect on the R_S_ (Yargeau et al., 2014).

If the concentration of the measured compound is sufficiently high, direct injection of the water being sampled in the analytical system is possible, simplifying the calibration procedure (Morin et al., 2013). Additionally, all interfering conditions, as pH, temperature, and conductivity, can be controlled during the calibration experiments. Laboratory calibration for drugs of abuse analysis using POCIS was already described by Yargeau et al., 2014.

Only a few studies reported R_S_ values for drugs of abuse, either obtained by *in situ* or laboratory calibration, as presented in Table 2. Also, the lack of standardization of the calibration procedures can result in significantly different R_S_ values for the same compound, as can be observed in the current literature.

**TABLE 2:** Target compounds, POCIS calibration, and sampling rates used to estimate water concentration of drug consumption biomarkers from POCIS

Compound	Sampling rates, R_S_ (L d ^−1^) (days or average)	POCIS calibration	POCIS type	POCIS sampling time	Estimation of drug use using POCIS	Sampling site	References
Cocaine	0.096 (av. 14 days); 0.087 (av. 28 days)	*In situ*	Pharm-POCIS (HLB 220 mg)	POCIS (*n* = 3) was replaced every 2 weeks during a 2 year-long period monitoring	Yes	WWTP in Oslo, Norway	Baz-Lomba et al. (2017)
BZE	0.039 (av. 14 days); 0.033 (av. 28 days)						
Methamfetamine	0.026 (av. 14 days); 0.026 (av. 28 days)						
Morphine	0.023 (av. 14 days); 0.021 (av. 28 days)						
Morphine	0.044 (14 days); 0.035 (av. 31 days)	*In situ*	Pharm-POCIS (HLB 200 mg)	POCIS (*n* = 3) was replaced every 2 weeks over a year-long period monitoring	Yes	WWTP in Oslo, Norway	Harman et al. (2011)
Amphetamine	0.125 (14 days); 0.094 (av. 31 days)						
MDMA	<0.097 (14 days); <0.118 (av. 31 days)						
Methamfetamine	0.128 (14 days); 0.102 (av. 31 days)						
OH-Meth	0.070 (14 days); 0.053 (av. 31 days)						
Cocaine	0.186 (14 days); 0.150 (av. 31 days)						
BZE^a^	0.083 (14 days)						
Cocaethylene	0.137 (14 days); 0.112 (av. 31 days)						
Cocaine	0.130 ± 0.036	Static laboratory-based calibration experiment, for 8 days	Pharm-POCIS (HLB 200 mg)	POCIS was deployed over a two-week period	No	WWTPs in Ontario and Quebec, Canada	Yargeau et al. (2014)
BZE	0.134 ± 0.011						
Amphetamine	0.201 ± 0.038						
MDA	0.288 ± 0.021						
Methamfetamine	0.231 ± 0.025						
MDMA	0.222 ± 0.013						
Ephedrine	0.123 ± 0.039						
Codeine	0.394 ± 0.049						
Dihydrocodeine	0.110 ± 0.041						
Morphine	0.261 ± 0.036						
Methadone	0.408 ± 0.147						
EDDP	0.532 ± 0.193						
Ketamine	0.197 ± 0.007	Bench-scale experiments with static exposure, for 3 days	Pharm-POCIS (HLB 200 mg)	POCIS (*n* = 3, per location) was deployed over a 2-week period	No	WWTP, at sites in the Grand River and in the DWTP in Ontario, Canada	Rodayan et al. (2016)
Fentanyl	0.390 ± 0.051						
Cocaine	0.13	Laboratory experiments conducted at water temperatures close to those in the cave systems (i.e., 26–28°C)	Pharm-POCIS (HLB)	POCIS (*n* = 3) was retrieved 28–32 days after deployment (depending on the site)	No	5 sites in flooded cave systems along the Caribbean coast of the Yucatan Peninsula in Mexico	Metcalfe et al. (2011)
BZE	0.13						
Amphetamine	0.26	Calculated theoretical uptake rates	Pharm-POCIS (HLB 200 mg)	POCIS was deployed for a 7-day exposure period, at each sampling location	No	WWTPs at Lincoln, Grand Island, Columbus, Hastings, and Omaha, in Nebraska, USA	Bartelt-Hunt et al. (2009)
Methamfetamine	0.22						

BZE, benzoylecgonine; DWTP, drinking water treatment plant; EDDP, 2-ethylidene-1,5-dimethyl-3,3-diphenylpyrrolidine; MDMA, 3,4-methylenedioxy-N-methylamphetamine; OH-Meth, hydroxymethamphetamine; THC-COOH, 11-nor-9-carboxy-THC; WBE, wastewater-based epidemiology; WWTP, wastewater treatment plant.

^a^ Uptake not linear after 14 days.

## Effect of Polar Organic Chemical Integrative Sampler Exposure Conditions on R_s_ Values

The R_S_ of a certain POCIS device is significantly affected by environmental conditions, like water flow (Alvarez et al., 2004; Bailly et al., 2013; Guibal et al., 2020), water temperature (Li et al., 2010), pH (Li et al., 2011), and biofouling (Harman et al., 2009).

The effect of water flow during POCIS sampling was evaluated by Guibal et al., 2020, for 44 pharmaceutical drugs, in a wide range of polarities. The calibration was performed at four different water flows: 0 (v_0_), 2-3 (v_1_), 6-7 (v_2_), and 20 (v_3_) cm s^−1^. Sampling rates were in the range of 0.040–0.218, 0.063–0.375, 0.062–0.408, and 0.075–0.539 L d^−1^ for v_0_, v_1_, v_2_, and v_3_, respectively. The authors concluded that an increase in water flow results in a decrease in the effective thickness of the water boundary layer at the POCIS membrane surface and, as a consequence, the increase in R_S_. A similar observation was previously described by Alvarez et al. (2004) that evaluated R_S_ of six micropollutants under quiescent (nonstirred) and turbulent (stirred) conditions. The adsorption of the evaluated chemicals was considered under aqueous boundary layer control, as shown by the increase in 4–9 times in R_S_ when water was agitated. The effect of water flow on R_S_ is dependent on the physicochemical properties of the investigated compounds. Bailly et al., 2013 found that an increase in water flow from 0.11 to 0.29 m s^−1^ did not affect R_S_ of sulfamethoxazole. Di Carro et al. (2014) evaluated R_S_ of several pesticides, pharmaceuticals, and chemicals by Pharm-POCIS and did not found differences at water flow rates in the range of 2–15.3 cm s^−1^.

The increase in water temperature influenced R_S_ of pharmaceuticals, personal care products, and endocrine disruptors adsorbed by POCIS, with an increase of up to two times when the temperature changed from 5 to 25°C (Li et al., 2010). Djomte et al. (2018) described a linear increase in R_S_ when increased in the range of 8–39°C in a constant water flow.

Li et al. (2011) studied the effect of pH on the R_S_ values on POCIS sampling. The R_S_ values of acidic pharmaceutical were reduced with the increase in pH from 3 to 9, whereas basic compounds presented the opposite trend. However, the observed R_S_ changes were with a three-fold range for the majority of the compounds. The dissolved organic matter (DOM) did not affect R_S_ in a relatively narrow range of value DOM values, from 3 to 5 mg L^−1^. The authors concluded that expected values of pH and DOM in natural water sources will result in small changes in R_S_ values.

Harman et al. (2009) fouled the POCIS before exposure to water containing the chemicals of interest. The fouling ranged from 0.2 to 2.8 g of dry weight dm^−2^, and exposure lasted for 6 weeks. Fouled POCIS adsorbed up to 55% more alkyl phenolic compounds than nonfouled POCIS.

Fouling can modify the mass transfer of the analyte, by increasing the thickness of the barrier or decreasing the size of membrane pores. Considering this possibility, Bailly et al., 2013 suggested that R_S_ values must be calculated using a matrix with organic content similar to the expected field conditions.

On the other hand, Rosen et al., 2018 did not found a relevant effect of biofouling on R_S_ of explosive compounds (2,4,6-trinitrotoluene and hexahydro-1,3,5-trinitro-1,3,5-triazine) when sampling was performed for up to 28 days. This behavior, different from the one observed for alkyl phenolic compounds, could be attributed to the higher polarity of the investigated chemicals.

Complementary studies are needed to clarify the impact of biofouling at POCIS adsorption of chemicals.

## Analytical Methods for the Measurements of Drugs of Abuse in Polar Organic Chemical Integrative Sampler

The measurement of drug consumption biomarkers in residual water requires the availability of sensitive analytical methods, usually after a concentration step. The concentration can be performed by a variety of extraction approaches, being solid-phase extraction the most commonly used strategy. When using POCIS, the sampling device can concentrate the analytes of interest in a very effective way. Oasis HLB^®^ is the more common sorbent used on POCIS for the determination of concentrations of drugs of abuse and its metabolites. This sorbent is highly versatile, being able to retain compounds with a wide range of polarities and acid-base properties, at variable pH ranges (Vazquez-Roig et al., 2013). After disassembling the device, the POCIS sorbent is usually transferred to an empty solid-phase extraction cartridge, washed with 10–20% methanol, and eluted using organic solvents (Harman et al., 2011; Baz-Lomba et al., 2017).

After extraction of the compounds of interest from the POCIS sorbent, analysis is usually performed using methods with mass spectrometric detection, particularly liquid-chromatography coupled to tandem mass spectrometry (LC-MS/MS) (Zuccato et al., 2005; van Nuijs et al., 2009a). LC-MS/MS is usually preferred once analytes in water are polar compounds, being amenable to liquid chromatographic separations without derivatization steps, and the technique also presents high sensitivity (Vazquez-Roig et al., 2013). Among the mass detector used in LC-MS/MS, triple quadrupoles are the most used due to its quantitative performance and robustness (Ort et al., 2014b; Thomas et al., 2012). The use of LC-MS/MS for the measurement of drug concentration in residual water requires the use of the deuterated internal standard to minimize matrix effects, which are usually significant when electrospray ionization sources are used (Castiglioni et al., 2006).

The uncertainty of the TWA concentrations estimated using POCIS was evaluated by Baz-Lomba et al. (2017). Two different confidence intervals were calculated, considering the precision of the R_S_ values obtained during *in situ* calibration and that R_S_ values could vary with a two-fold interval. The uncertainty (U) was estimated for five pharmaceutical compounds, using the following equation:

$$U=\frac{CV}{\surd n}$$ [8]

The uncertainty ranged from ±35.4% for atenolol to ±43.1% for metoprolol. The uncertainties were attributed mainly due to the variability during the *in situ* calibration of the POCIS.

## Previous Reports of Polar Organic Chemical Integrative Sampler Use for Wastewater-Based Epidemiology of Drugs of Abuse

Despite the attractiveness of POCIS use in terms of cost and versatility, once a few studies had used this sampling strategy in WBE studies for drugs of abuse. Table 2 presents an overview of studies that employed POCIS for the measurement of drugs of abuse concentrations. However, in the text below, only reports that used POCIS measurements for WBE of drugs of abuse will be discussed.

An early report applied POCIS for the evaluation of drug consumption in the city of Oslo, in Norway (Harman et al., 2011). In this study, the sampling campaign lasted for a whole year, and several temporal trends in drug consumption in the evaluated population were identified. Besides drugs of abuse, authors also monitored concentrations of cetirizine, an antihistaminic drug mostly used during spring months. In fact, cetirizine concentrations in POCIS samples collected during spring, when a high incidence of seasonal rhinitis is observed, were more than two-fold the levels measured in POCIS collected during the winter. Authors considered this finding as an indication of the POCIS capability of detecting time-related patterns of drug use in a monitored population. The same study reported peaks of MDMA consumption during a popular student celebration in Norway, as well as fluctuations in the consumption of COC and amphetamine over the year, with prominent peaks on summer and winter, usually associated with holidays. Variation in the estimated drug consumption over the year can also be associated to the variable availably of the different drugs. The authors estimated COC consumption based on the TWA concentrations of BZE, resulting in consumed amounts of 20–70 mg day^−1^ 1.000 inh^−1^. When using TWA concentration of COC for the estimation of the drug consumption, values were in the range of 310–2,800 mg day^−1^ 1.000 inh^−1^. The authors of this study concluded that consumption based on COC concentrations was more accurate when compared with studies performed in other European cities using active sampling. Measuring BZE in relation to COC is the preferred approach since COC can be present in wastewater without having been used and because COC exhibits significant degradation. However, due to the nonlinear absorption kinetics of BZE presented in their work, measuring COC may be more appropriate when using POCIS. In the case of amphetamine and methamphetamine, average daily consumption was estimated to be in the range of 190 mg day^−1^ 1.000 inh^−1^ and 400 mg day^−1^ 1.000 inh^−1^, respectively.

Baz-Lomba et al. (2017) also made a WBE study in Oslo, with POCIS sampling being performed continuously for two years. Using BZE concentrations, the average COC consumption during the years of 2012 and 2013 was 120 mg day^−1^ 1.000 inh^−1^, which was considered as adequately concordant with estimations made using BZE levels obtained after an active composite sampling campaign, of 152 mg day^−1^ 1.000 inh^−1^, reported by SCORE group in 2015. Average methamphetamine consumption during the years of 2012 and 2013 was estimated as 263 mg day^−1^ 1.000 inh^−1^, also in concordance with the active sampling estimations.

There are a few report of POCIS R_S_ of illicit drugs in the literature. As summarized in Table 2, reported R_S_ is very variable even for the same compound. *In situ* determined R_S_ for COC was reported in the range of 0.096–0.186 L d^−1^ and laboratory calibration reports presented the value of 0.13 L d^−1^. Also for BZE, a similar pattern is observed, with *in situ* determined R_S_ of 0.039 and 0.083 L d^−1^, and laboratory calibration reports described R_S_ value of 0.13 L d^−1^. Considerable differences can also be noted for morphine (*in situ* R_S_ of 0.023–0.044 L d^−1^; laboratory R_S_ of 0.261 L d^−1^) and methamphetamine (*in situ* R_S_ of 0.026 and 0.128 L d^−1^; laboratory R_S_ of 0.231 L d^−1^). The widely variable of POCIS R_S_ values shows that these values are highly dependent of the experimental calibration conditions and also characterize the semiquantitative nature of the drug consumption estimation using POCIS in WBE. Baz-Lomba et al. (2017) performed a WBE study in the same location than Harman et al., 2011, using POCIS sampling, which were calibrated *in situ*. The R_S_ values described by Baz-Lomba et al., 2017 were almost the half for cocaine, BZE, and morphine and about five times lower for methamphetamine. One possible explanation for these differences could be the impact of the water flow rate around the passive samplers, with higher turbulence leading to increased R_S_ values due to the reduction of the water boundary layer over the POCIS membrane (Guibal et al., 2020).

Detailed analytical data are not available in several manuscripts summarized in Table 2. Liquid chromatography coupled to mass spectrometry was used in all studies, either with tandem quadrupole (Bartelt-Hunt et al., 2009; Harman et al., 2011; Metcalfe et al., 2011; Yargeau et al., 2014), ion trap (Rodayan et al., 2016), or time-of-flight (Baz-Lomba et al., 2017) detectors. Bartelt-Hunt et al. (2009) estimated a limit of detection lower than 1 ng ml^−1^, which relates to an absolute amount of 1 ng recovered from the POCIS. Also, recovery of target compounds was checked by analysis of fortified blanks spiked with known amounts of each compound, averaging 123 ± 30%. Rodayan et al. (2016) measured drug concentrations in wastewater, with limits of quantification between 0.48 and 8.4 ng L^−1^, according to the measured analyte. The analyte recovery from POCIS was higher than 80%. The concentrations of some analytes measured in grab samples were lower than TWA estimated from POCIS. In some cases, analytes were detected or quantifiable in POCIS but not in the corresponding grab samples, such as which illustrates the value of passive sampling for concentrating trace contaminants to detectable levels and the importance of effective sampling strategies. Yargeau et al. (2014) collected wastewater specimens both using POCIS and automatic composite sampling. Authors reported that methamphetamine, dihydrocodeine, and oxycodone were detected on POCIS but not in all composite samples. These findings support previous studies showing that POCIS may accumulate drugs to detectable levels when these compounds are not detectable in grab or composite samples of wastewater. Harman et al. (2011) reported quantification limits for target compounds in POCIS 0.5 and 5 ng POCIS^−1^ (morphine and methamphetamine were exceptions with limits of 10 and 50 ng POCIS^−1^, respectively). Baz-Lomba et al. (2017) reported recovery for all tested compounds in their study from the HLB POCIS sorbent in the range of 72–118%.

**FIGURE 2:**
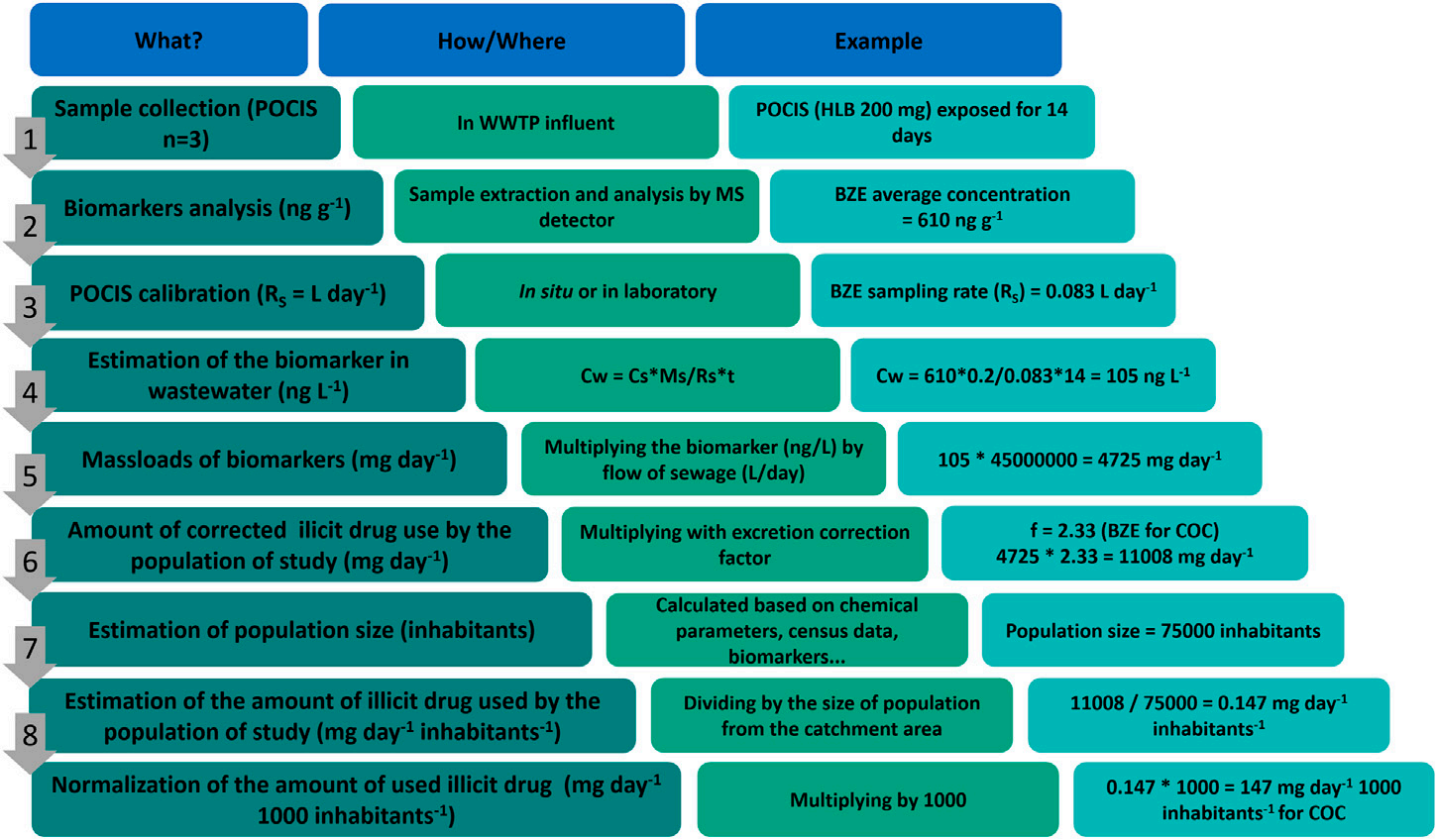
Outline exemplifying the estimation of consumption of COC by wastewater analysis using POCIS as a sampling tool.

Figure 2 outlines all steps for a WBE study for drugs of abuse consumption using POCIS, from sampling to consumption estimation. In this example, an estimate of COC consumption after BZE concentration measurement is exemplified.

## Conclusion

Wastewater-based epidemiology is a useful tool to detect illicit drug use of a population in real-time, allowing effective health and law-enforcement actions. The application of wastewater-based epidemiology requires that representative samples are obtained in practical and effective way. An attractive, adaptable, and low-cost alternative for sampling of biomarkers of drug consumption in residual water is the use of POCIS. Average biomarkers concentration in residual water can be estimated using POCIS, particularly for compounds presenting linear accumulation kinetics on the sorbent. To date, only few studies applied POCIS in WBE and more studies are required before the use of this sampling strategy can be considered as standard. However, considering the cost of composite active samplers and also the operational requirements of these equipment, the use of POCIS is very attractive to allow WBE long-term studies in limited resources settings, even considering its semiquantitative nature.
